# Mulberry based zinc nano-particles mitigate salinity induced toxic effects and improve the grain yield and zinc bio-fortification of wheat by improving antioxidant activities, photosynthetic performance, and accumulation of osmolytes and hormones

**DOI:** 10.3389/fpls.2022.920570

**Published:** 2022-09-27

**Authors:** Muhammad Umer Chattha, Tahira Amjad, Imran Khan, Muhammad Nawaz, Muqarrab Ali, Muhammad Bilal Chattha, Hayssam M. Ali, Rehab Y. Ghareeb, Nader R. Abdelsalam, Saira Azmat, Lorenzo Barbanti, Muhammad Umair Hassan

**Affiliations:** ^1^Department of Agronomy, University of Agriculture Faisalabad, Faisalabad, Pakistan; ^2^Department of Agricultural Engineering, Khwaja Fareed University of Engineering and Information Technology, Rahim Yar Khan, Pakistan; ^3^Department of Agronomy, Muhammad Nawaz Shareef University of Agriculture, Multan, Pakistan; ^4^Department of Agronomy, Faculty of Agricultural Sciences, University of the Punjab, Lahore, Pakistan; ^5^Department of Botany and Microbiology, College of Science, King Saud University, Riyadh, Saudi Arabia; ^6^Department of Plant Protection and Biomolecular Diagnosis, Arid Lands Cultivation Research Institute, The City of Scientific Research and Technological Applications, New Borg El Arab, Egypt; ^7^Department of Agricultural Botany, Faculty of Agriculture, Saba Basha, Alexandria University, Alexandria, Egypt; ^8^Agriculture Extension and Adaptive Research, Department of Agriculture, Government of the Punjab, Punjab, Pakistan; ^9^Department of Agricultural and Food Sciences, University of Bologna, Bologna, Italy; ^10^Research Center Ecological Sciences, Jiangxi Agricultural University, Nanchang, China

**Keywords:** bio-fortification, hormones, osmolytes, nutrients, salinity stress, wheat, zinc

## Abstract

Salinity stress (SS) is a challenging abiotic stress that limits crop growth and productivity. Sustainable and cost effective methods are needed to improve crop production and decrease the deleterious impacts of SS. Zinc (Zn) nano-particles (NPs) have emerged as an important approach to regulating plant tolerance against SS. However, the mechanisms of SS tolerance mediated by Zn-NPs are not fully explained. Thus, this study was performed to explore the role of Zn-NPs (seed priming and foliar spray) in reducing the deleterious impacts of SS on wheat plants. The study comprised different SS levels: control, 6 and 12 dS m^−1^, and different Zn-NPs treatments: control, seed priming (40 ppm), foliar spray (20 ppm), and their combination. Salinity stress markedly reduced plant growth, biomass, and grain yield. This was associated with enhanced electrolyte leakage (EL), malondialdehyde (MDA), hydrogen peroxide (H_2_O_2_), sodium (Na), chloride (Cl) accumulation, reduced photosynthetic pigments, relative water contents (RWC), photosynthetic rate (Pn), transpiration rate (Tr), stomata conductance (Gs), water use efficiency (WUE), free amino acids (FAA), total soluble protein (TSP), indole acetic acid (IAA), gibberellic acid (GA), and nutrients (Ca, Mg, K, N, and P). However, the application of Zn-NPs significantly improved the yield of the wheat crop, which was associated with reduced abscisic acid (ABA), MDA, H_2_O_2_ concentration, and EL, owing to improved antioxidant activities, and an increase in RWC, Pn, Tr, WUE, and the accumulation of osmoregulating compounds (proline, soluble sugars, TSP, and FAA) and hormones (GA and IAA). Furthermore, Zn-NPs contrasted the salinity-induced uptake of toxic ions (Na and Cl) and increased the uptake of Ca, K, Mg, N, and P. Additionally, Zn-NPs application substantially increased the wheat grain Zn bio-fortification. Our results support previous findings on the role of Zn-NPs in wheat growth, yield, and grain Zn bio-fortification, demonstrating that beneficial effects are obtained under normal as well as adverse conditions, thanks to improved physiological activity and the accumulation of useful compounds. This sets the premise for general use of Zn-NPs in wheat, to which aim more experimental evidence is intensively being sought. Further studies are needed at the genomic, transcriptomic, proteomic, and metabolomic level to better acknowledge the mechanisms of general physiological enhancement observed with Zn-NPs application.

## Introduction

Abiotic stresses undermine crop production and threaten global food security. Salinity stress (SS) is an emerging stress factor as it hampers crop growth and productivity, and global food security (Zafar et al., 2020). Globally, more than 33% of soils are salt-affected, and this figure is expected to increase up to 50% by the end of 2050 (Gopalakrishnan and Kumar, 2020). Salinity stress has a harmful impact on plant metabolic processes and imposes osmotic injuries due to salt-induced ionic toxicity (Egamberdieva et al., 2019; Kamran et al., 2019). SS also disturbs nutrient homeostasis, water uptake, stomata opening, and chlorophyll synthesis. It causes a significant reduction in photosynthetic performance (Zafar et al., 2021). A higher salt concentration in the plant root zone reduces the water uptake, which in turn causes salinity induced osmotic stress, altering normal plant functions (Sonam et al., 2014).

Salinity stress also reduces the uptake of the major cationic nutrients (Ca^2+^, K^+^, and Mg^2+^), and increases the uptake of undesirable ions (Na and Cl; Sharaf et al., 2016). The increase in such ions reduces membrane stability and increases ion toxicity, and decreases photosynthetic efficiency (Zafar et al., 2021) due to stomata closing, reduced chlorophyll synthesis, and carbon dioxide (CO_2_) assimilation, resulting in stunted plant growth (Shahid et al., 2011). Salinity stress also increases the production of reactive oxygen species (ROS), which damage protein, deoxyribonucleic acid (DNA), lipids, meristematic and photosynthetic activities, destruct chlorophyll, and disturb nutritional homeostasis (Sarker et al., 2018; Hassan et al., 2019a; Aslam et al., 2021; Qari et al., 2022; Rehman et al., 2022; Batool et al., 2022a,b). Moreover, SS also damages chloroplast ultra-structure and photo-system efficiency, electron transport and negatively affects gas exchange (Hurtado et al., 2019). Additionally, SS reduces the number of leaves per plant, root and shoot growth, and biomass production. As these changes can cause huge yield losses (Ramadan et al., 2019; Khan et al., 2022), appropriate measures to mitigate the adverse effects of SS on plants are urgently needed.

More than 40 million tons of agrochemicals are used annually globally for crop production, and this quantity will double by the end of 2050 to feed the 9.5 billion people (Kah et al., 2019). Thus, it is mandatory to introduce new techniques that can effectively achieve sustainable crop productivity. Nanotechnology has emerged as an important field and its use has substantially increased in many areas of life including agriculture. Plants can benefit from nano-particles (NPs) as nutrient sources that can also increase their tolerance against different stresses (Raliya et al., 2017). Various kinds of NPs are used in diverse fields of life; however, zinc (Zn) NPs are widely used in agriculture and different other sectors (Adrees et al., 2021).

Zinc is an important micro-nutrient for plants and has beneficial effects on plant growth; however, it also has some toxic effects at a higher concentration. Zn is involved in protein and chlorophyll synthesis and is also needed for metabolic turnover and enzymatic activities (Saravanakkumar et al., 2016). Not surprisingly, deficiency of Zn has a negative effect on plants, thus, the use of ZnO-NPs is an interesting approach to fulfilling the Zn needs of plants (Du et al., 2019). Approximately, 550 tons Zn-NPs are produced annually across the globe for different uses (Alabdallah and Alzahrani, 2020). The NPs of Zn are considered to be safe as compared to other metals (Kim et al., 2020). Zn nano-particles exert benefits on soil fertility, and crop productivity and they are an important source of Zn, which is an important nutrient for plant growth and protection from different stresses (Rossi et al., 2019; Esper Neto et al., 2020; Salim and Raza, 2020). The use of NPs could minimize the use of toxic and harsh chemicals for plant growth, helping to avoid damage to our environment and soil health (Maroufpour et al., 2020).

The application of NPs is reported to positively influence plant response to SS (Elsheery et al., 2020; Zulfiqar and Ashraf, 2021). NPs induce profound impacts on physiological and biochemical processes (Etesami et al., 2021). Zn-NPs regulate the SS tolerance in plants by improving membrane stability, anti-oxidant activities, ionic homeostasis, and gene expression (Noohpisheh et al., 2021). However, these effects can vary depending on plant species, environmental conditions, size and shape of NPs, and their rate of application (Etesami et al., 2021). Wheat is the staple food of more than 50% of people across the globe; however, it is considered only a moderately salt tolerant species (Spano and Bottega, 2016). Wheat is an important source of both macro and micro nutrients for humans. Among micro nutrients, Zn is an important nutrient needed for humans and plants; however, in both cases, Zn deficiency is an issue of major concern. Bio-fortification is an important strategy globally, to improve the grain Zn concentration and fulfill human needs (Hassan et al., 2019b). This approach involves the use of both agronomic and breeding practices to improve grain Zn (Hassan et al., 2021). Breeding approaches are costly and time demanding (Hassan et al., 2021), and agronomic approaches may be a quick solution to solve this problem (Hassan et al., 2021). The agronomic approach involves the use of micronutrients by different methods to enhance grain Zn concentration (Chattha et al., 2017; Hassan et al., 2019b).

Salt stress significantly reduces the growth and productivity of the wheat crop. Various practices are used globally to overcome the effects of SS on wheat crops. Nonetheless, the effect of Zn-NPs has not been sufficiently studied. Although a few studies are available about the effect of Zn-NPs on the performance of the wheat crops grown under SS, they have not fully explored the mechanism linked to Zn-NP induced SS tolerance. There is no information available related to the effect of Zn-NPs produced through a green approach to wheat growth, yield, and grain Zn concentration under SS.

We hypothesize that the application of Zn-NPs could alleviate the toxic effects of SS by improving antioxidant activities, physiological functioning, and accumulation of stress protecting hormones and osmolytes. The present study aimed to determine the impact of Zn-NPs on physiological attributes, antioxidant enzymes, ionic homeostasis, osmo-regulating compounds, yield, and grain Zn bio-fortification of wheat crops grown under SS.

## Materials and methods

### Experimental setup

The experiment was performed at the wire house of the Department of Agronomy, University of Agriculture Faisalabad, Pakistan, to determine the effects of zinc (Zn) nano-particles (NPs) applied by seed priming and foliar spray on the wheat crop under SS. During this period (November 2019 to April 2020), the average temperature of 17.6°C was recorded, whereas average humidity and total rainfall were observed at 71.1% and 28.4 mm. Salt sensitive variety of wheat, Punjab 2011 was used as a test crop. The study comprised three SS levels: control, 6 and 12 dS m^−1^, and three patterns of Zn-NPs treatment: control, seed priming (40 ppm Zn), foliar spray (20 ppm Zn), and the combination of seed priming (40 ppm) + foliar spray (20 ppm). The soil used in the experiment was taken from an experimental field using a spade. Afterward, debris and weeds were removed, and the soil was sieved and properly stored. The soil was recognized as sandy loam with pH 7.82, organic matter (OM) 0.83%, total nitrogen (N) 0.041%, available phosphorus (P) 6.7 mg kg^−1^, and exchangeable potassium (K) 158 mg kg^−1^.

Pots with a capacity of 8 kg and a diameter of 28 cm were then filled with a mixture of soil and sand (9:1). The desirable levels of soil salinity were achieved by adding Na_2_CO_3_ and NaCl. The concentration of salts was calculated with the formula below:


$$\mathrm{NaCl}\;\mathrm{required}\;\left(g/\mathrm{kg}\right)\frac{TSS\times 58.5\times \mathrm{saturation}\;\left(\%\right)}{100\times 100}$$

In this equation, TSS is total soluble salts that were measured with following formula:


$$TSS=\left(EC2-EC1\right)\times 10$$

EC_1_ was the original electrical conductivity (EC) of soil, whereas EC_2_ was the EC as per our treatments. The soil paste was prepared by adding the distilled water and allowed for 2 h to reach equilibrium. This mixture was then filtered with filter paper to obtain the extract. The soil mixture was then oven dried at 105°C and soil saturation was measured with the following formula:


$$\mathrm{saturation}\;\left(\%\right)\frac{\mathrm{loss}\;\mathrm{in}\;\mathrm{soil}\;\mathrm{weight}\;\mathrm{on}\;\mathrm{drying}}{\mathrm{weight}\;\mathrm{of}\;\mathrm{soil}\;\mathrm{after}\;\mathrm{drying}}\times 100$$

To attain SS levels 6 and 12 dS m^−1^, NaCl was added at the rate of 1.179 and 2.58 g/kg, whereas Na_2_CO_3_ was added as; 0.023 and 0.50 g/kg, respectively.

### Crop husbandry and preparation of Zn-NPs

Eight seeds were sown in each pot. For seed priming, 40 mg Zn-NPs were dissolved in 1,000 of distilled water, and seeds were soaked in this solution for 6 h before they were dried and sown in pots. For foliar application, 20 mg Zn-NPs were dissolved in 1,000 ml of water to prepare the solution for foliar spray. N and P fertilizers such as urea and di-ammonium phosphate (DAP) were applied at the rate of 1.46 and 1.56 g pot^−1^, respectively. The pots were regularly visited and irrigated as per crop needs. The irrigation was applied to each based on visual observations with the help of a hand sprayer. The weeds occurring in pots were manually pulled and no insect pest attack was observed; therefore, no pesticide was applied. The leaves of mulberry plants were washed with distilled water; after that, they were oven dried and milled to make powder. The powder samples (20 g) were mixed in distilled water (100 ml) and boiled at 75°C for 45 min (Saravanakkumar et al., 2016). After that, the extract was obtained and stored at 4°C. Later on, Zn-NPs were prepared by adding 20 ml extract to 80 ml of ZnSO_4_.7H_2_O. The solution was then sonicated for 1 h at 60°C, and the change in color to yellow showed the formation of Zn-NPs (Eya’ane Meva et al., 2016).

### Data collection

#### Growth parameters and gas exchange characteristics

Three plants were chosen at random from each pot to determine growth traits. After that, the roots and shoots of the selected plants were oven dried to determine dry weights. Photosynthetic rate, (Pn), transpiration rate (Tr), stomata conductance (gs), and water use efficiency (WUE) were measured by using a portable infra-red gas analyzer (IRGA) from randomly selected plants from each pot. All these characteristics were recorded at 10–12 am at maximum sunlight.

#### Photosynthetic pigments

For determination of chlorophyll contents, 0.5 g of fresh leaves were taken and ground in 80% solution of 5 ml acetone. The ground sample was centrifuged for 3 min. A clear supernatant was collected after centrifugation and absorbance were measured using a spectrophotometer at three wavelengths, i.e., 645 nm (Chl a), 663 nm (Chl. b), and 470 nm (carotenoids) as per methods of Arnon (1949). Subsequently, we took 5 ml of 50 mM potassium phosphate buffer, and an additional 0.5 g of fresh leaves were ground. The material was then centrifuged, the supernatant was placed in a cuvette, and the sample absorbance was measured using a spectrophotometer at 600 nm to determine anthocyanin contents (Kubo et al., 1999).

#### Relative water contents and electrolyte leakage

One gram of fresh leaves was taken and weighed on an electrical scale to determine fresh weight. After that, they were dipped in distilled water (H_2_O) for 24 h. Then leaves were removed from the water, excess water was wiped out and leaves were weighed again to determine turgid weight. Afterward, the leaves were sun-dried and packed in small paper bags and oven dried at 70°C for 24 h to determine the dry weight. Relative water content (RWC) was determined with this formula: RWC = (FW-DW)/(TW-DW) × 100.

Half a gram of fresh leaf sample (chopped into small pieces) was dipped in distilled water for half an hour, and EC1 was measured by using an EC meter. EC2 was recorded by heating the samples in a water bath at 90°C for 50 min. The final value of electrolyte leakage (EL; %) was determined with this formula:


$$\mathrm{EL}\%=\left(EC1÷EC2\right)\times 100.$$

#### Anti-oxidant activities

Test tubes containing 100 μl of plant extract, H_2_O_2_ (5.9 mM), and 1,000 μl buffer solution were prepared, and we noted absorbance at 240 nm (Aebi, 1984). The technique of Zhang (1992) was used for the determination of peroxidase (POD) activity. We took 0.1 ml H_2_O_2_ added 2.7 ml phosphate buffer and shook it well. Then, 0.1 ml of guicol and 0.1 ml of enzyme extract were added and shaken again, and absorbance was recorded with a spectrophotometer at 470 nm. The mixture containing 100 μl enzyme extract, 100 μl ascorbate (7.5 mM), 100 μl H_2_O_2_ (300 mM), and 2.7 ml potassium buffer (25 mM) was prepared, and absorbance was taken at 290 nm for determining ascorbate peroxidase (APX) activity (Nakano and Asada, 1981). Lastly, for determination of POD; 400 μl H_2_O_2_, 25 ml buffer, 100 μl Tritoen, 50 μl nitroblue tetrazolium chloride (NBT), 50 μl sample, and 50 μl riboflavin were taken, and absorbance was recorded at 560 nm (Zhang, 1992).

#### H2O2 and MDA determination

The technique of Velikova et al. (2000) was used to determine the quantity of H_2_O_2_ in the sample. We ground 0.25 g of plant leaves in 5 ml of 5% TCA and centrifuged them for 5 min, and the extract was then collected. Then, 1 ml crude sample, 1 ml potassium iodide (1 M), and 100 ml phosphate buffer were added, and absorbance was recorded at 600 nm. For malondialdehyde (MDA) determination, a 0.5 g plant sample was ground in 5 ml of TCA and centrifuged for 15 min. Afterward, the mixture was heated for 30 min at 100°C and cooled rapidly and absorbance was recorded at 532 nm (Rao and Sresty, 2000).

#### Osmo-regulating compounds and hormones

A quantity of 0.5 g plant material was ground in 5 ml potassium buffer, centrifuged for 5 min at 10,000 rpm, and the supernatant was collected. Then, 1 ml 2% ninhydrin solution and 1 ml 10% pyridine solution were added to test tubes containing the extract and warmed for 30 min in a water bath at 90°C. Distilled water was added to maintain volume at 15 ml, and absorbance was recorded with a spectrophotometer at 570 nm (Hamilton and Van Slyke, 1943). In the case of TSP, 0.5 g of plant sample was ground in potassium phosphate buffer (50 mM) and centrifuged for 15 min and 1 ml of supernatant was added with 2 ml of Bradford reagent. This solution was left for 15 min at room temperature, and absorbance was read at 590 nm (Bradford, 1976). In the case of soluble sugars1–2 ml of supernatant was placed on the prism of the refractometer and brix percentage was noted. For proline content, a 0.5 g plant sample was extracted with 10 ml of 3% sulpho-salicylic acid and centrifuged at 10,000 rpm for 10 min. Acid-ninhydrin was then added to the supernatant and placed in a water bath (30 min), and absorbance was recorded at 520 nm (Bates et al., 1973).

For the determination of plant hormones, a 0.5 g plant sample was ground in 80% methanol (2 ml) with 40 mg of butylated hydroxytoluene. Lastly, the obtained extract was dissolved in 0.1% gelatin (pH 7.5), and 0.1% Tween-20 consisting of 2 ml of phosphate-buffered and the concentration of indole-3-acetic acid (IAA) and abscisic acid (ABA) was determined with the method of Weiler et al. (1981). For determination of gibberellic acid (GA) content, 0.1 g plant was taken and extracted by using 3 ml of 96%, ethanol, and absorbance was taken at 254 nm (Berríos et al., 2004).

#### Ion concentrations

The plant samples were collected, washed, oven dried (65°C), and ground to make powder. After that, 0.5 g samples were digested in the acid mixture (HCl:HNO_3_, 1:2) at 180°C for 10 min. The concentration of Na and K was determined by a flame photometer (Sultan et al., 2021). Moreover, for the determination of Cl content, a chloride analyzer was used on the prepared extract. Lastly, the ground wheat grains were taken and digested in the acid mixture (HCl:HNO_3_, 1:2), then absorbance was recorded using atomic absorption spectrophotometer to determine grain Zn concentration (Sultan et al., 2021).

#### Determination of yield traits

Randomly selected three plants from each pot were taken to determine the number of tillers, spike length, grains/spike, and spikelets/spike. Then, plant samples were manually harvested, threshed, and grains were separated from the spikes. 1,000-grain weight was recorded by randomly taking 1,000 seeds.

#### Experimental design and data analysis

The experiment was conducted in a completely randomized design. The 3 × 3 factorial combinations of SS and Zn-NPs per three replicates made a total of 27 pots. The data on different traits were submitted to the ANOVA for the main factors and their interaction. The LSD test at *p* ≤ 0.05 was used for determining significant differences among means (Steel et al., 1997). Moreover, figures were created by using the SigmaPlot-8 software.

## Results

### Growth traits

Salinity stress caused a marked reduction in growth traits of wheat (Table 1). The root and shoot length and biomass were significantly decreased under strong SS (12 dSm^−1^) as compared to moderate SS (6 dS m^−1^). In response to this, the application of Zn-NPs significantly mitigated the salinity induced toxic effects and appreciably improved the growth traits and biomass production. However, the application of Zn-NPs by seed priming and foliar spray markedly improved the growth traits as compared to the other methods of single Zn-NPs application. Seed priming (40 ppm) + foliar spray (20 ppm) of Zn-NPS appreciably improved the shoot length (SL: 30.9%), shoot fresh weight (SFW: 54.4%), shoot dry weight (SDW: 48.3%), root fresh weight (RFW: 14.9%), root dry weight (RDW: 19.5%), and leaves per plant (LPP: 12%) as compared to control under strong SS (12 dS m^−1^; Table 1).

**Table 1:** Effect of Zn-NPs application on growth attributes of wheat plants grown under salinity stress.

**Salinity stress**	**Zn-NPs**	**SL (cm)**	**SFW (g)**	**SDW (g)**	**RL (cm)**	**RFW (g)**	**RDW (g)**	**LPP**
**Control**	**CK**	71.97d ± 0.94	6.24 cd ± 0.47	3.45d ± 0.015	17.73c ± 0.57	3.88 cd ± 0.038	1.86c ± 0.030	9.33bc ± 0.33
	**SP**	78.12b ± 0.49	7.02b ± 0.26	5.45b ± 0.018	21.03b ± 0.95	4.22b ± 0.050	2.14a ± 0.032	10.00b ± 0.16
	**FS**	75.07c ± 0.45	6.58bc ± 0.29	4.45c ± 0.014	19.80b ± 0.31	4.01bc ± 0.045	1.96b ± 0.023	10.00b ± 0.17
	**SP + FS**	83.37a ± 1.19	8.64a ± 0.50	5.75a ± 0.012	22.77a ± 0.92	4.53a ± 0.16	2.22a ± 0.024	11.33a ± 0.21
**6 dS m** ^**−1**^	**CK**	59.65i ± 0.55	3.60gh ± 0.14	1.75j ± 0.021	13.17fgh ± 0.45	3.47e ± 0.058	1.57e ± 0.044	8.67 cd ± 0.25
	**SP**	68.67e ± 0.65	5.58de ± 0.21	2.65f ± 0.025	15.90de ± 0.30	3.75d ± 0.045	1.69d ± 0.038	9.33bc ± 0.27
	**FS**	63.77 fg ± 0.32	5.15e ± 0.12	2.25 g ± 0.019	14.67ef ± 0.38	3.71d ± 0.139	1.61de ± 0.04	9.33bc ± 0.22
	**SP+ FS**	72.75d ± 0.54	5.94 cd ± 0.34	3.25e ± 0.039	17.30 cd ± 0.90	3.80 cd ± 0.036	1.81c ± 0.036	9.67b ± 0.41
**12 dS m** ^**−1**^	**CK**	50.02j ± 1.25	3.27 h ± 0.28	1.45 l ± 0.029	10.09i ± 0.28	2.75 h ± 0.025	1.28 g ± 0.039	8.33d ± 0.24
	**SP**	62.66gh ± 0.90	4.95ef ± 0.39	1.95j ± 0.034	12.17gh ± 0.34	3.08 fg ± 0.039	1.43f ± 0.020	9.33bc ± 0.29
	**FS**	61.40hi ± 0.81	4.30 fg ± 0.28	1.65 k ± 0.036	11.73 h ± 0.65	2.86gh ± 0.060	1.36 fg ± 0.032	8.33d ± 0.35
	**SP+ FS**	65.50f ± 0.72	5.05ef ± 0.32	2.15 h ± 0.039	13.50 fg ± 0.59	3.16f ± 0.035	1.53e ± 0.017	9.33bc ± 0.21

CK, control; SP, seed priming (40 ppm); FP, foliar application (20 ppm); SL, shoot length; SFW, shoot fresh weight; SDW, shoot dry weight; RL, root length; RFW, root fresh weight; RDW, root dry weight; and LPP, leaves per plant. Data reported indicate means ± SE (*n* = 4). Different letters indicate significant differences at *p* < 0.05.

### Photosynthetic pigments and leaf gas exchange characteristics

**Figure 1:**
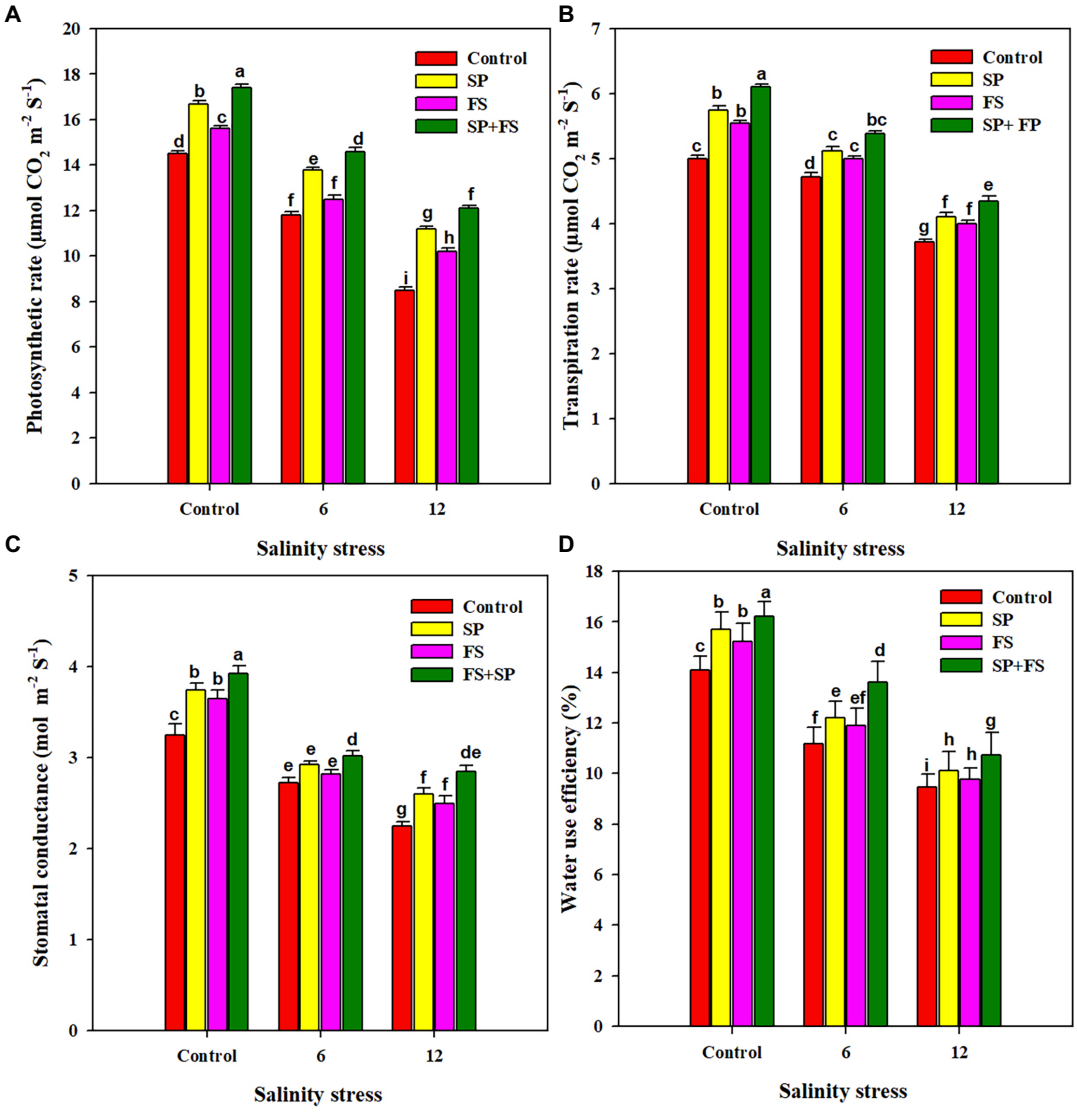
Effect of Zn-NPs on photosynthetic rate **(A)**, transpiration rate **(B)**, stomata conductance **(C)**, and water use efficiency **(D)** of wheat plants grown under salinity stress. Data depicted indicate means ± SE (*n* = 4). Different letters indicate significant differences at *p* < 0.05.

Salinity stress induced a deleterious impact on the photosynthetic pigments (Table 2). The increasing concentration of salts in the growing medium up to 12 dS m^−1^ significantly reduced the chlorophyll, carotenoid, and anthocyanin contents (Table 2). Nonetheless, the application of Zn-NPs markedly improved the synthesis of all the aforementioned photosynthetic pigments under control and the two SS levels. The combined application of Zn-NPs by seed priming and foliar spray was shown the top performer, and it significantly increased the chlorophyll-a, chlorophyll-b, carotenoid, and anthocyanin by a respective 66.7, 76.6, 54.2, and 93.8% under strong SS (12 dS m^−1^), as compared to control (Table 2). SS also induced a distinctive impact on all the gas exchange parameters (Figure 1). The increased salt concentration in the growing medium up to 12 dS m^−1^ decreased the Pn, Tr, Gs, and WUE by 60.6, 34.4, 44.4, and 49.2%, respectively, as compared to the control (Figure 1). The application of Zn-NPS appreciably improved all the gas exchange parameters under SS (Figure 1). However, the combined use of Zn-NPs by seed priming and foliar spray was proved the top performer in improving the Pn, Tr, Gs, and WUE, as compared to other methods of Zn-NPs application and control (Figure 1).

**Table 2:** Effect of Zn-NPs application on photosynthetic pigments and anthocyanin contents of wheat plants grown under salinity stress.

**Salinity stress**	**Zn-NPs**	**Chlorophyll a (mg/g FW)**	**Chlorophyll b (mg/g FW)**	**Carotenoids (mg/g FW)**	**Anthocyanin (mg/g FW)**
**Control**	**CK**	0.38d ± 0.012	1.41d ± 0.021	0.52d ± 0.0054	8.31d ± 0.45
	**SP**	0.54b ± 0.019	1.70b ± 0.029	0.60b ± 0.0064	9.83b ± 0.72
	**FS**	0.46c ± 0.012	1.56c ± 0.026	0.56c ± 0.0042	9.16c ± 0.21
	**SP + FS**	0.62a ± 0.014	1.85a ± 0.016	0.64a ± 0.0039	10.51a ± 0.56
**6 dS m** ^**−1**^	**CK**	0.17 g ± 0.0056	0.73i ± 0.014	0.27 k ± 0.0043	4.45j ± 0.49
	**SP**	0.22f ± 0.017	1.12f ± 0.010	0.45f ± 0.0026	6.95f ± 0.92
	**FS**	0.14 g ± 0.021	0.97 g ± 0.091	0.41 g ± 0.0028	6.28 g ± 0.44
	**SP + FS**	0.30e ± 0.0031	1.26e ± 0.022	0.49e ± 0.0032	7.63e ± 0.71
**12 dS m** ^**−1**^	**CK**	0.03hi ± 0.0037	0.47j ± 0.010	0.24 l ± 0.0054	2.89 k ± 0.69
	**SP**	0.04hi ± 0.0012	0.68i ± 0.014	0.33i ± 0.0042	4.92i ± 0.44
	**FS**	0.02I ± 0.0019	0.52j ± 0.016	0.30j ± 0.0032	4.50j ± 0.48
	**SP + FS**	0.05 h ± 0.0011	0.83 h ± 0.018	0.37 h ± 0.0043	5.60 h ± 0.32

CK, control; SP, seed priming (40 ppm); and FP, foliar application (20 ppm). Data reported indicate means ± SE (*n* = 4). Different letters indicate significant differences at *p* < 0.05.

### Relative water contents and oxidative stress markers

**Figure 2:**
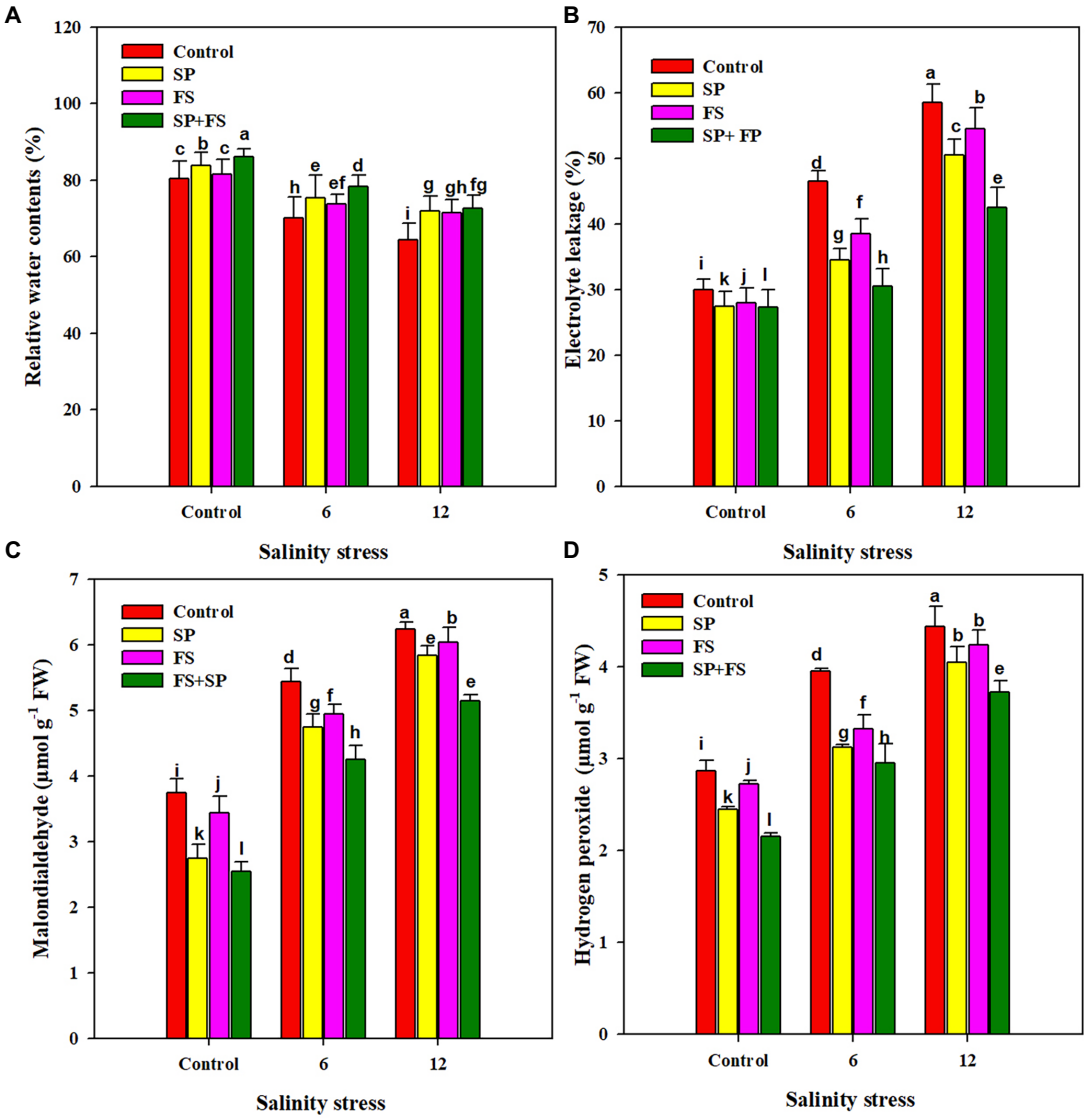
Effect of Zn-NPs on relative water content **(A)**, electrolyte leakage **(B)**, malondialdeide **(C)**, and hydrogen peroxide **(D)** of wheat plants grown under salinity stress. Data depicted indicate means ± SE (*n* = 4). Different letters indicate significant differences at *p* < 0.05.

The RWC of wheat plants grown under SS was significantly decreased, as compared to control plants (Figure 2). An RWC reduction of 14.8 and 25.1%, respectively, was recorded at moderate (6 dS m^−1^) and strong SS (12 dS m^−1^), as compared to the control. Zn-NPs markedly improved the RWC of wheat plants under both control and SS conditions. The application of Zn-NPs by seed priming + foliar spray appreciably improved the leaf RWC, as compared to control and other methods of single Zn-NPs application (Figure 2). SS significantly increased the EL, and the MDA and H_2_O_2_ accumulation in wheat plants (82, 66.9, and 54.7%, respectively) under a SS level of 12 dS m^−1^ (Figure 2). The application of Zn-NPs significantly reduced the concentration of all the oxidative stress markers. In this context, the combined application of Zn-NPs by seed priming and foliar spray was once more shown as the top performed, and it significantly reduced the EL (37.6 and 52.5%), and the MDA (21.1 and 28.0%) and H_2_O_2_ content (19.0 and 33.9%) under the respective moderate and strong SS (Figure 2).

### Anti-oxidant activities

**Figure 3:**
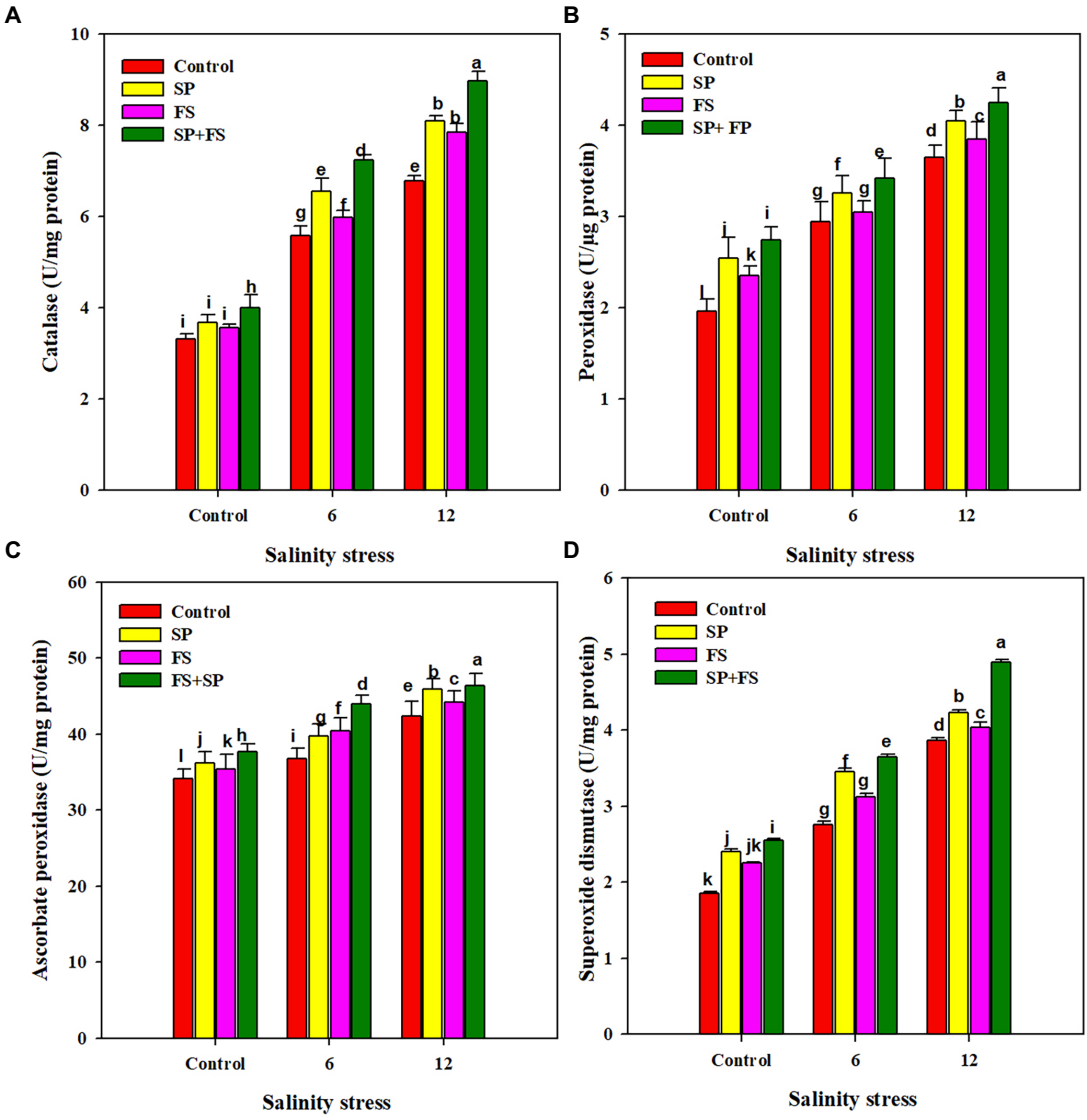
Effect of Zn-NPs on antioxidant activities: catalase **(A)**, peroxidase **(B)**, ascorbate peroxidase **(C)**, and superoxide dismutase **(D)** of wheat plants grown under salinity stress. Data depicted indicate means ± SE (*n* = 4). Different letters indicate significant differences at *p* < 0.05.

The activities of APX, CAT, POD, and SOD were markedly increased under SS. The application of Zn-NPs by all the methods significantly increased the activities of all antioxidants (Figure 3). However, the combined use of Zn-NPs with seed priming and foliar spray was portrayed as the top performer, and it appreciably increased the activity of APX, CAT, POD, and SOD by 14.4, 32.4, 16.4, and 26.4%, respectively, under strong SS as compared to control (Figure 3).

### Osmo-regulating compounds and hormones

A distinctive response of osmoregulating compounds and hormones was recorded under SS and with the application of Zn-NPs. SS significantly reduced the concentration of TSP and FAA, and maximum reduction was recorded under strong SS (Table 3). Moreover, the concentration of total soluble sugars (TSS) and proline showed a significant increase under SS (Table 3). The application of Zn-NPs significantly increased the accumulation of TSP, FAA, TSS, and proline, and the combined use of Zn-NPs by seed priming and foliar spray was shown to the top performed, and it significantly increased the accumulation of all these protective osmolytes (Table 3).

**Table 3:** Effect of Zn-NPs application on hormones and osmolytes contents of wheat plants grown under salinity stress.

**Salinity stress**	**Zn-NPs**	**TSP**	**FAA**	**SS**	**Proline**	**IAA**	**GA**	**ABA**
**Control**	**CK**	7.86c ± 0.054	1.19d ± 0.046	10.51j ± 0.16	0.64f ± 0.037	18.73 g ± 0.39	45.83 h ± 0.66	35.40 cd ± 0.59
	**SP**	8.29b ± 0.068	1.32b ± 0.023	11.39i ± 0.09	0.68ef ± 0.017	21.37ef ± 0.58	48.77 fg ± 0.44	32.17de ± 0.93
	**FS**	8.08bc ± 0.044	1.26bc ± 0.017	11.10i ± 0.05	0.64f ± 0.013	20.47 fg ± 0.52	46.30gh ± 0.68	34.13de ± 1.28
	**SP+ FS**	8.84a ± 0.014	1.43a ± 0.014	11.97 h ± 0.12	0.71ef ± 0.023	23.43de ± 0.60	50.30ef ± 0.96	31.43e ± 1.01
**6 dSm** ^**−1**^	**CK**	6.83ef ± 0.080	1.08e ± 0.009	11.99 h ± 0.10	0.74de ± 0.039	21.53ef ± 0.67	48.17fgh ± 1.01	41.23b ± 1.38
	**SP**	7.24d ± 0.054	1.11e ± 0.001	14.17f ± 0.28	0.81 cd ± 0.034	25.53d ± 1.13	52.63e ± 1.04	34.90cde ± 0.83
	**FS**	7.00e ± 0.089	1.08e ± 0.017	13.13 g ± 0.14	0.76de ± 0.020	22.10ef ± 0.86	49.17f ± 0.86	38.53bc ± 1.12
	**SP+ FS**	7.93c ± 0.036	1.20 cd ± 0.039	14.83e ± 0.18	0.85bc ± 0.017	28.37c ± 0.98	58.60 cd ± 1.58	32.20de ± 0.25
**12 dSm** ^**−1**^	**CK**	6.20 h ± 0.017	0.88f ± 0.021	15.97d ± 0.14	0.82 cd ± 0.029	30.07c ± 0.20	57.73d ± 0.63	47.40a ± 1.16
	**SP**	6.63 fg ± 0.076	0.96f ± 0.023	17.32b ± 0.14	0.92ab ± 0.049	34.43ab ± 0.52	64.17b ± 0.97	41.93b ± 1.29
	**FS**	6.40gh ± 0.092	0.89f ± 0.045	16.75c ± 0.07	0.85bc ± 0.017	33.03b ± 1.18	60.60c ± 0.52	45.70a ± 0.79
	**SP+ FS**	6.92e ± 0.075	1.10e ± 0.017	18.33a ± 0.29	0.99a ± 0.037	36.87a ± 1.48	67.97a ± 1.44	39.53b ± 0.44

CK, control; SP, seed priming (40 ppm); FP, foliar application (20 ppm); TSP, total soluble proteins; FAA, free amino acids; SS, soluble sugars; IAA, indole-3-acetic acid; GA, gibberellic acid; and ABA, abscisic acid. Data reported indicate means ± SE (*n* = 4). Different letters indicate significant differences at *p* < 0.05.

Moreover, an increase in IAA, GA, and ABA accumulation was seen under SS. The application of Zn-NPs also increased the accumulation of IAA and GA, whereas it markedly reduced the accumulation of ABA. The combined application of Zn-NPs by seed priming and foliar spray increased the IAA and GA concentration by 22.6 and 17.7%, respectively, whereas it reduced the ABA concentration by 19.5% under strong SS (Table 3).

### Nutrient concentration in plant parts

The concentration of all the ions was significantly affected by the SS and application of Zn-NPs. SS markedly increased the accumulation of Na and Cl in plant roots and shoots while it reduced the accumulation of K in plant parts (Table 4). Zn-NPs reduced Na and Cl concentration, while they increased the accumulation of K in plant parts grown under SS (Table 4). Likewise, SS also reduced the concentration of Ca, Mg, N, P, and Zn in wheat roots and shoots, and the maximum reduction was noted under strong SS (Tables 4, 5). Zn-NPs appreciably improved the concentration of Ca, Mg, N, P, and Zn in the two plant portions. However, the combined application of Zn-NPs as seed priming and foliar spray significantly increased the concentration of Ca, Mg, N, P, and Zn, as compared to the single seed priming or foliar spray application (Tables 4, 5).

**Table 4:** Effect of Zn-NPs application on nutrient contents in root and shoot of wheat plants grown under salinity stress.

**Salinity stress**	**Zn-NPs**	**Root-Na**^**+**^	**Shoot-Na**^**+**^	**Root-K**^**+**^	**Shoot-K**^**+**^	**Root-Cl**^**−**^	**Shoot-Cl**^**−**^	**Root-Ca**	**Shoot-Ca**
**mg g**^**−1**^ **DW**									
**Control**	**CK**	2.57a ± 0.083	2.78f ± 0.11	21.34bc ± 0.49	26.63c ± 0.58	3.19 g ± 0.13	2.71 g ± 0.032	55.90de ± 0.90	66.13 cd ± 1.11
	**SP**	2.19a ± 0.022	2.34f ± 0.049	24.83a ± 1.24	34.77a ± 0.91	2.66 g ± 0.11	2.42 g ± 0.015	60.80b ± 1.00	71.43b ± 0.58
	**FS**	2.29a ± 0.057	2.58f ± 0.059	22.63b ± 0.87	30.43b ± 0.53	2.86 g ± 0.064	2.61 g ± 0.026	58.77bc ± 0.38	68.50bc ± 0.51
	**SP + FS**	2.15a ± 0.026	2.29f ± 1.22	26.83a ± 0.94	36.87a ± 0.93	2.60 g ± 0.029	2.25 g ± 0.023	63.33a ± 1.11	76.47a ± 1.32
**6 dSm** ^**−1**^	**CK**	20.15b ± 0.078	24.60bc ± 0.54	13.40 fg ± 0.64	19.97f ± 0.38	20.40d ± 0.38	16.50d ± 0.57	47.87 g ± 0.52	56.67gh ± 0.52
	**SP**	15.60d ± 0.871	21.23de ± 0.72	17.53de ± 0.58	23.47d ± 0.58	17.13e ± 0.72	13.87f ± 0.23	54.80e ± 1.23	63.14de ± 1.26
	**FS**	18.53bc ± 0.33	23.07 cd ± 1.38	15.10f ± 0.32	22.30ef ± 0.62	18.47de ± 0.23	15.63de ± 0.15	50.55f ± 0.32	59.30 fg ± 0.63
	**SP + FS**	13.00e ± 0.75	19.83e ± 1.49	22.47b ± 1.15	26.87c ± 0.85	14.70f ± 0.50	12.90f ± 0.63	57.80 cd ± 0.41	68.40bc ± 1.33
**12 dSm** ^**−1**^	**CK**	24.83a ± 1.23	31.13a ± 0.92	12.57 g ± 0.27	16.20 g ± 0.70	32.73a ± 0.36	25.60a ± 0.30	40.27i ± 0.46	48.53i ± 0.67
	**SP**	20.40b ± 1.12	24.07bc ± 0.38	15.50ef ± 0.50	21.20f ± 1.15	23.03c ± 0.43	18.90c ± 0.48	47.77 g ± 0.87	55.57 h ± 1.18
	**FS**	22.97a ± 0.67	26.03b ± 1.11	13.67 fg ± 0.43	17.50f ± 0.43	26.43b ± 0.54	21.30b ± 0.58	43.10 h ± 0.91	50.93i ± 0.74
	**SP + FS**	17.40 cd ± 0.49	22.33cde ± 0.73	19.37 cd ± 0.48	23.43d ± 0.61	17.73e ± 0.77	15.31e ± 0.42	51.27f ± 0.55	60.47ef ± 0.46

CK, control; SP, seed priming (40 ppm); FP, foliar application (20 ppm); Na, sodium; K, potassium; Cl, chloride; and Ca, calcium. Data reported indicate means ± SE (*n* = 4). Different letters indicate significant differences at *p* < 0.05.

**Table 5:** Effect of Zn-NPs application on nutrient contents in root and shoot of wheat plants grown under salinity stress.

**Salinity stress**	**Zn-NPs**	**Root-Mg**	**Shoot-Mg**	**Root-N**	**Shoot-N**	**Root-P**	**Shoot-P**	**Root-Zn**	**Shoot-Zn**
**mg g**^**−1**^ **DW**									
**Control**	**CK**	40.70d ± 0.36	50.77c ± 0.42	8.60d ± 0.077	14.50 cd ± 0.63	6.24c ± 0.071	8.12d ± 0.015	17.83b ± 0.28	24.13c ± 0.97
	**SP**	45.83b ± 0.93	56.23b ± 1.22	9.94b ± 0.12	16.70ab ± 0.60	6.93b ± 0.14	8.61b ± 0.032	21.40a ± 0.64	27.60b ± 0.64
	**FS**	43.03c ± 0.82	54.27b ± 0.91	9.29c ± 0.14	15.53bc ± 0.56	6.47c ± 0.62	8.41c ± 0.096	21.43a ± 0.52	27.70b ± 0.25
	**SP+ FS**	49.43a ± 0.49	59.10a ± 0.82	10.68a ± 0.29	17.73a ± 0.43	7.29a ± 0.13	9.12a ± 0.092	22.40a ± 1.27	32.67a ± 0.67
**6 dSm** ^**−1**^	**CK**	34.47 g ± 0.64	40.17f ± 0.88	6.70gh ± 0.10	11.88 fg ± 0.22	5.27efg ± 0.09	7.07f ± 0.060	14.03d ± 0.45	20.37d ± 0.38
	**SP**	40.43d ± 0.55	46.50d ± 0.55	7.27f ± 0.18	13.85de ± 0.33	5.78d ± 0.043	7.48e ± 0.043	16.07c ± 0.56	23.17c ± 0.19
	**FS**	37.70ef ± 0.40	43.37e ± 0.99	7.05 fg ± 0.078	12.87ef ± 0.30	5.45e ± 0.026	7.30e ± 0.078	16.17c ± 0.34	23.28c ± 0.58
	**SP+ FS**	43.63c ± 0.47	51.51c ± 0.66	7.97e ± 0.27	14.50 cd ± 0.63	6.28c ± 0.14	7.95d ± 0.026	17.46bc ± 0.4	24.37c ± 0.67
**12 dSm** ^**−1**^	**CK**	32.27 h ± 0.76	35.47 h ± 0.56	5.30 k ± 0.15	8.29i ± 0.21	4.87 h ± 0.11	5.96j ± 0.023	10.80e ± 0.35	15.60f ± 0.78
	**SP**	36.10 fg ± 0.64	40.47f ± 0.62	5.94ij ± 0.13	10.72gh ± 0.35	5.17 fg ± 0.044	6.60 h ± 0.097	13.70d ± 0.55	17.97e ± 0.38
	**FS**	34.50 g ± 0.61	37.80 g ± 0.36	5.59jk ± 0.41	9.73hi ± 0.29	4.99gh ± 0.046	6.23i ± 0.045	13.80d ± 0.40	18.10e ± 0.89
	**SP+ FS**	39.47de ± 0.92	45.47de ± 0.55	6.26hi ± 0.12	12.70ef ± 0.49	5.42ef ± 0.12	6.80 g ± 0.087	14.47d ± 0.68	18.80de ± 0.49

CK, control; SP, seed priming (40 ppm); and FP, foliar application (20 ppm). Mg, magnesium; N, nitrogen; and P, phosphorus. Data reported indicate means ± SE (*n* = 4). Different letters indicate significant differences at *p* < 0.05.

### Yield traits and grain Zn bio-fortification

**Figure 4:**
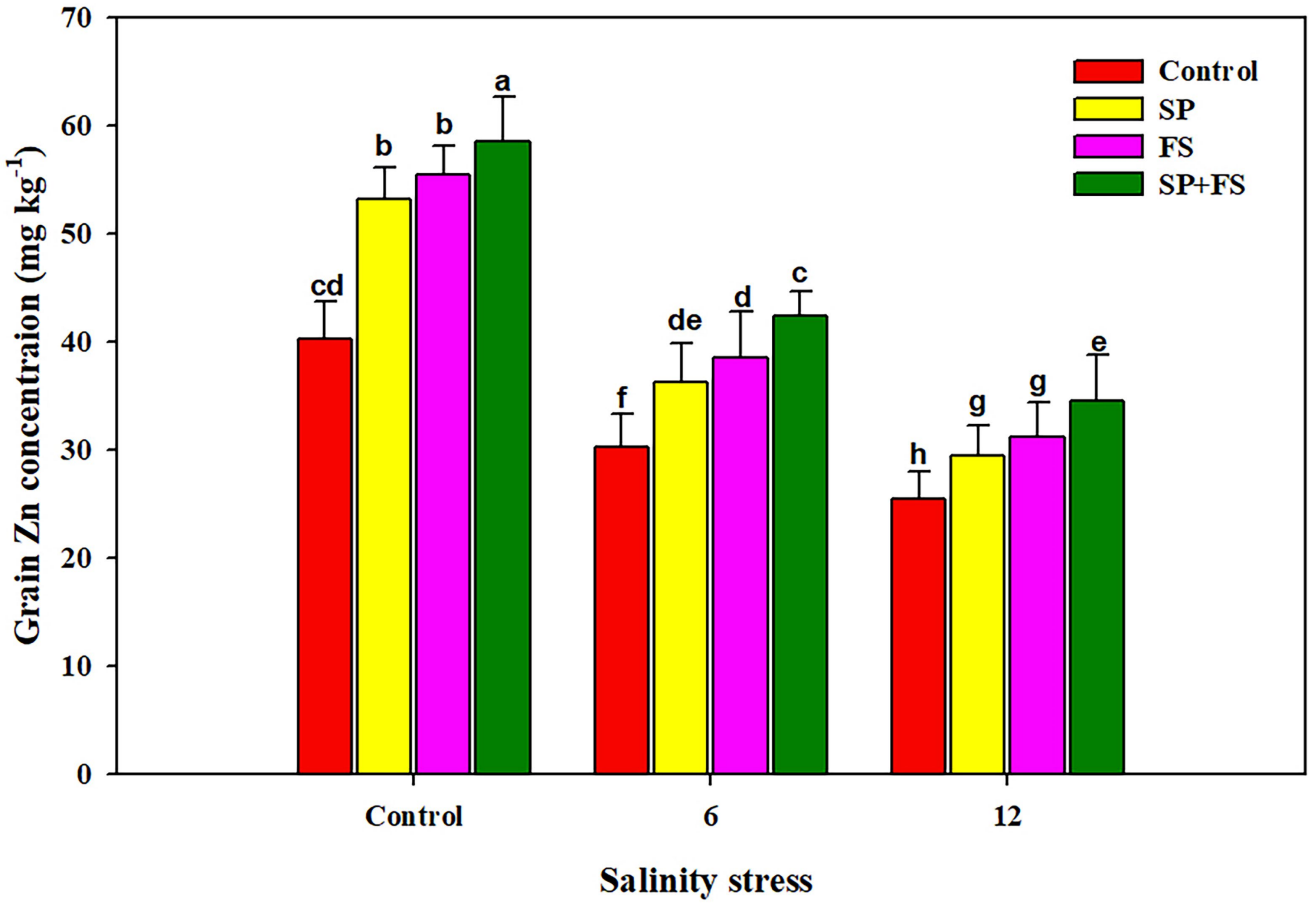
Effect of Zn-NPs on grain Zn concentration of wheat plants grown under salinity stress. Data depicted indicate means ± SE (*n* = 4). Different letters indicate significant differences at *p* < 0.05.

Salinity stress and application of Zn-NPs significantly influenced wheat yield traits (Table 6). SS determined sharp reductions of 23.2, 71.1, and 64.8%, respectively, in spike length, spikelets/spike, and grains/spike (Table 6). The application of Zn-NPs mitigated this reduction and significantly increased the aforementioned yield traits under control and SS conditions. Moreover, SS also significantly reduced the 1,000 grain weight and grain yield/pot. The maximum reduction in 1,000 grain weight and grain yield/pot was recorded in 12 dS m^−1^ SS as compared to 6 dS m^−1^ SS (Table 6). The combined use of Zn-NPs by seed priming and foliar spray markedly improved the 1,000 grain weight (32.6 and 12.2%, respectively) and grain yield/pot (23.2 and 71.1%, respectively) under moderate and strong SS (Table 6). The increase in salinity significantly decreased the grain Zn concentration (Figure 4), in response to which, the application of Zn-NPs significantly improved the grain Zn concentration. Nonetheless, the Zn-NPs application by combined seed priming and foliar spray significantly increased the grain Zn contents as compared to the other two methods of single Zn-NPs application, and control (Figure 4).

**Table 6:** Effect of Zn-NPs application on yield traits of wheat plants grown under salinity stress.

**Salinity stress**	**Zn-NPs**	**SL (cm)**	**SLPS**	**GPS**	**TGW (g)**	**GY/pot (g)**
**Control**	**CK**	6.88d ± 0.18	28.50d ± 0.57	36.50c ± 0.74	34.50d ± 0.79	35.83c ± 0.92
	**SP**	7.97b ± 0.15	36.50b ± 0.88	46.75a ± 1.29	42.50b ± −0.88	40.27b ± 1.03
	**FS**	8.92c ± 0.21	32.50c ± 0.92	40.50b ± 0.88	41.50b ± 1.22	38.40b ± 1.06
	**SP+ FS**	9.90a ± 0.56	40.50a ± 1.12	48.50a ± 1.46	46.50a ± 1.45	42.70a ± 0.96
**6 dSm** ^**−1**^	**CK**	5.92gh ± 0.19	12.50 h ± 1.20	20.50f ± 0.88	24.95gh ± 0.75	25.63 fg ± 0.43
	**SP**	7.35de ± 0.22	24.50ef ± 1.76	28.50d ± 1.66	31.50e ± 0.68	29.37e ± 0.62
	**FS**	6.55efg ± 0.32	25.00def ± 0.88	24.50e ± 1.26	27.62f ± 1.05	27.57ef ± 0.52
	**SP+ FS**	7.77d ± 0.30	27.00de ± 0.59	34.50c ± 1.58	33.00de ± 0.86	32.57d ± 0.85
**12 dSm** ^**−1**^	**CK**	5.52 h ± 0.48	10.50 h ± 1.08	13.25 g ± 1.73	22.87 h ± 0.58	16.10i ± 0.46
	**SP**	6.20fgh ± 0.26	18.25 g ± 1.52	21.75ef ± 1.25	25.90 fg ± 0.72	21.03 h ± 0.74
	**FS**	6.12fgh ± 0.24	14.25 h ± 0.66	21.25ef ± 1.28	25.22 g ± 1.65	18.77j ± 0.49
	**SP+ FS**	6.92ef ± 0.17	21.75 fg ± 1.14	22.25ef ± 1.49	25.65 fg ± 1.41	23.47 g ± 0.60

CK, control; SP, seed priming (40 ppm); and FP, foliar application (20 ppm). SL, spike length; SLPS, spikelets/spike; GPS, grains/spike; TGW, 1,000 grain weight; and GY, grain yield. Data reported indicate means ± SE (*n* = 4). Different letters indicate significant differences at *p* < 0.05.

## Discussion

Soil salinity is a serious abiotic stress that limits crop productivity across the globe. Wheat is considered a salt sensitive crop, and SS can cause huge growth and yield losses in wheat crops. Therefore, increasing crop production on salt affected soils is a serious challenge (Zhanwu et al., 2014). The present study was planned to avert the effects of SS on the wheat crop by exogenous application of Zn-NPs. SS significantly decreased plant growth (Table 1). Excessive concentration of salts in the growing medium causes ionic toxicity and osmotic imbalance, and disturbs nutrient and water uptake and plant photosynthetic efficiency. All this results in reduced growth and biomass productivity (Lv et al., 2013). The high Na concentration (Table 4) reduced K^+^ accumulation which in turn serves to reduce cell turgid pressure; therefore, cells become rigid and cannot reach their maximum size (Lv et al., 2013; Abdelgawad et al., 2016; Fatma et al., 2021). However, the application of Zn-NPs significantly improved wheat growth (Table 1). The application of Zn maintained membrane integrity, improved K^+^ uptake (Table 4), gas exchange parameters (Figure 1), antioxidants (Figure 3), and reduced the uptake of Na, resulting in overall improvement of plant growth. The application of Zn-NPs improved the auxin biosynthesis (Table 3), chlorophyll production, nutrient uptake (Table 4), and reduced the salinity induced stomata closure by decreasing ABA (Table 3), which also contributed to significant increases in growth and biomass productivity (Khaghani and Saffari, 2016; Rajput et al., 2018).

Chlorophyll plays a key role in photosynthesis, and a reduction in chlorophyll content reduces the photosynthetic rate and the accumulation of photosynthates in plant organs (Taiz and Zeiger, 2006). SS increases the activities of enzymes involved in chlorophyll degradation, therefore, contributes to decreasing chlorophyll content (Rao and Rao, 2013). SS also reduces the uptake of Mg^2+^ ions, which are considered to be building blocks of the chlorophyll molecule; therefore, a decrease in Mg^2+^ uptake causes a marked reduction in chlorophyll synthesis (Fatma et al., 2021). Moreover, salinity induced oxidative stress damages the chloroplast structure and causes chlorophyll degradation, owing to an increase in chlorophyllase enzyme activity (Latef and Chaoxing, 2011; Latef et al., 2014). However, Zn-NPs reduced the negative impacts of SS and enhanced chlorophyll content (Table 2). The application of Zn-NPs reduced the activities of chlorophyll degradation enzymes and resultantly increased the chlorophyll content and subsequent plant growth under SS (Mehrabani et al., 2018; Sturikova et al., 2018). Zn-NPs can penetrate the plant cells by leaf stomata and improve the plant metabolic activities and synthesis of photosynthetic pigments under stress conditions (Venkatachalam et al., 2017; Alabdallah and Alzahrani, 2020). Zn is crucial for the formation of *protochlorophyllide*, it positively affects chloroplasts and repairs PS-II, and therefore, causes an increase in photosynthetic pigments under SS (Hänsch and Mendel, 2009; Salama et al., 2019). In our experiment, the application of Zn also positively regulated the Mg^2+^ uptake, which is considered to be an important component of chlorophyll structure; hence, Zn application increased the chlorophyll content of wheat plants. SS also caused a significant decrease in anthocyanin content (Table 2); however, Zn-NPs increased the anthocyanin contents (Table 2). Anthocyanin regulates ROS accumulation and maintains photosynthetic efficiency (Xu and Rothstein, 2018). Therefore, Zn-NPs mediated increase in anthocyanin content might protect the photosynthetic apparatus and therefore improve the chlorophyll content and plant photosynthetic efficiency.

Salinity stress significantly reduced stomata conductance, transpiration rate, intercellular CO_2,_ and WUE (Figure 1). The reduction in stomata conductance under SS generally occurs due to reductions in vapor pressure, leaf turgor potential, and root generated chemical signals (Chaves et al., 2009). Moreover, a reduction in stomata conductance (Figure 1) significantly reduced the transpiration owing to closing stomata. Additionally, salinity induced accumulation of undesirable ions (Na and Cl), decreases the rate of photosynthesis by damaging the photosynthetic apparatus (Xu and Zhou, 2008; Biswal et al., 2011; Xu and Rothstein, 2018). However, the application of Zn-NPs significantly improved the entire gas exchange parameters under SS, in accordance with the results obtained by Torabian et al. (2016), who found significant improvements in the aforementioned gas exchange parameters with the application of Zn-NPs. Zn application regulated the stomata movements by maintaining higher K^+^ (Table 4) in guard cells, which maintain higher stomata conductance under SS. Moreover, Zn-NPs also reduced the ABA accumulation (Table 3) that might favor stomata opening and, therefore, maintain higher Tr and WUE in wheat plants under SS. The membrane damage is measured by MDA content, and in the present study, SS caused a significant increase in MDA accumulation (Figure 2). Salinity induced ion toxicity that leads to a higher accumulation of MDA owing to membrane damage. The excessive Na participates in ROS production, which causes damage to cellular membranes and increases the MDA accumulation under SS (Sultan et al., 2021). Zn-NPs alleviated the membrane damage by increasing in anti-oxidant activities (Figure 3), therefore reducing the MDA accumulation under SS (Hussein et al., 2017). RWC was considerably decreased under SS Figure 2); however, the application of Zn contrasted this reduction in leaf RWC (Figure 2). SS induced osmotic stress which reduces water uptake, therefore, reduces the leaf RWC (Kim et al., 2020; Fatma et al., 2021). The present increase in RWC by Zn-NPs may be attributed to improved membrane integrity, stomata conductance, and Tr (Figure 1), as well as an increase in water uptake due to better root growth, which helps the plants to maintain higher leaf RWC. The present study indicates that SS caused a significant increase in Na accumulation in plant parts, while it reduced the K accumulation. The accumulation of Na in plant cells causes ionic imbalance because above a modest concentration Na is a toxic inorganic ion (Chen et al., 2012). The increase in SS increased the Na^+^ accumulation which inhibited the K uptake (Table 4), with a consequential effect on stomata guard cells (Munns and Tester, 2008; Saddiq et al., 2021). Because of the low concentration of external protons, SS reduces the ability of Na/K antiporters to exclude excessive Na which, therefore, increases the Na accumulation at the expense of K accumulation in plant cells (Munns and Tester, 2008). However, the application of Zn-NPs significantly reduced the Na accumulation while maintaining a higher K concentration in all plant parts (Table 4). Possibly, Zn application maintained the structural integrity and controlled the permeability of root cell membranes; therefore, reduced the excessive Na uptake and consequently reduced the Na accumulation while maintaining higher K accumulation in wheat plants.

The results indicated that SS significantly up-regulated activities of APX, CAT, POD, and SOD; application of Zn-NPs also improved antioxidant activities (Figure 3). SOD is considered to be an important enzyme that decomposes the O^2−^radical into H_2_O_2_, and SOD is also considered the first line of defense under SS. These findings are in agreement with the previous findings of El-Esawi et al. (2018) and who also found a significant increase in anti-oxidant activities (APX, CAT, POD, and SOD) under SS. In the present work, the application of Zn-NPs significantly improved antioxidant activities, which is consistent with the findings of Rizwan et al. (2019), who also found a significant increase in anti-oxidant activities with application of Zn-NPs under SS. The application of Zn in the form of NPs improves the expression of anti-oxidant genes (SOD and CAT), and therefore maintains the higher activity of SOD and CAT, and minimizes the salinity induced oxidative stress (Pavithra et al., 2017). Zn deficiency reduced Zn-SOD activity; however, Zn supply restores enzymatic activity because Zn is a structural component of Zn-SOD (Cakmak, 2000). Zn is also needed for the higher activity of CAT which also plays an important role in H_2_O_2_ detoxification (Wani et al., 2013). The application of Zn decomposes O_2_; therefore, it maintains higher CAT activity under SS (Sharaf et al., 2016). The application of Zn also increases the activity of APX, which is attributed to the ability of Zn to facilitate the synthesis of this antioxidant (Cakmak, 2000; Amini et al., 2016). Lastly, an increase in CAT activity following the application of Zn-NPs also indicates that the Zn-NPs improve CAT activity to mitigate the adverse effects of SS.

A higher concentration of proline under SS suggests that plants accumulate proline as a defensive osmolyte against SS (Hmidi et al., 2018). SS also reduced the IAA concentration, which could be due to over-accumulation of nitric oxide (NO) (Liu et al., 2015). It is well known that ABA increases under SS and handles stomata closure; nonetheless, this trade-off reduces plant growth and photosynthesis due to limitations in gas exchange (Fujita et al., 2005; Fatma et al., 2021). The increase in ABA under SS induced stomata closure and reduced the Tr (Figure 1), thereby reducing WUE and overall photosynthetic efficiency (Tanaka et al., 2013). Plants also accumulate soluble sugars. Which appreciably maintains the turgor pressure and enhances the membrane stability under SS by scavenging ROS (Hussein et al., 2017). Zn-NPs maintained a higher proline and soluble sugar accumulation (Table 3). This increase can be attributed to an increase in the activities of enzymes involved in proline biosynthesis. The application of Zn-NPs maintained a higher IAA level. Zn is known to be a co-enzyme needed for tryptophan synthesis that is a precursor to the formation of IAA (Waraich et al., 2011). Therefore, Zn induces an increase in IAA that can be attributed to an increase in tryptophan synthesis under SS. The increase in IAA improves root growth, which facilitates nutrient and water uptake; therefore, IAA improves plant growth under SS conditions (Waraich et al., 2011). The application of Zn-NPs also improved GA concentration, which also leads to a substantial increase in growth (Table 1) by regulating plant osmotic balance (Jamil et al., 2018). The application of Zn-NPs significantly reduced the ABA accumulation, which could be an important mechanism of Zn induced SS tolerance in plants. However, more studies at the metabolomic, proteomic, and transcriptomic level are needed to explore how Zn-NPs application reduces the accumulation of ABA under SS.

The results indicated that SS caused a significant reduction in yield and yield traits of wheat plants. SS induced ionic, osmotic, and oxidative stresses which damage the photosynthetic apparatus and alter plant physiological functioning as stomata closure, reduction in leaf expansion, and altered gas exchange parameters, which in turn reduce the final productivity (Zahra et al., 2018; Javaid et al., 2019). The higher concentration of Na causes water deficiency, which is a major reason for a substantial reduction in crop yield across the globe (Iqbal et al., 2019; Yadav et al., 2020). However, in this experiment, the Zn application significantly improved the yield and yield traits under SS, thanks to improved plant physiological functioning, gas exchange parameters (Figure 1), metabolic activities, chlorophyll synthesis, antioxidant activity, and accumulation of osmolytes (Hassan et al., 2020). SS significantly reduced the grain Zn content, which could be attributed to excessive Na concentration in the growing medium reducing the Zn uptake. However, Zn-NPs determined a noticeable bio-fortification of wheat grain. The application of Zn-NPs reduced Na uptake while maintaining a higher uptake of Zn, which contributed to a significant improvement in grain Zn content. Moreover, the results indicated that different methods of Zn-NPs application significantly affected the grain Zn content; nonetheless, the combined application of seed priming + foliar spray significantly improved the grain Zn concentration (Figure 4). The foliar applied Zn markedly increased the grain Zn content, although a little quantity of Zn is applied in this method as compared to soil application (Cakmak et al., 2010). The foliar applied Zn maintains higher Zn availability within plant tissues during reproductive stages; therefore, it substantially increased the grain Zn concentration (Chattha et al., 2017).

## Conclusion

Salinity stress markedly affected the wheat plant by altering the photosynthetic performance, nutrient uptake, osmolyte, and hormone accumulation. However, the application of Zn-NPs offset the salinity induced toxic effects, and improved wheat growth and final yield. The application of Zn-NPs showed a reduction in EL, MDA, H_2_O_2_, and ABA accumulation, in exchange for an increase in photosynthetic pigments, leaf gas exchange parameters, hormone, and osmolyte accumulation, and antioxidant activities, resulting in a substantial improvement in plant growth functions. Further, the beneficial role of Zn-NPs was evidenced by the restricted entry of toxic ions (Na and Cl), and improved uptake of major nutrients as the sodium contrasting cations Ca, K, and Mg, plus N and P. Based on our results, the application of Zn-NPs can be seen an effective approach to improve wheat growth, final yield and obtain grain Zn bio-fortification under salinity stress as well as normal conditions. Therefore, it is evinced that Zn-NPs use, especially the double seed priming and foliar spray application, could be a winning strategy under many circumstances, although further evidence is needed in support of this. Additionally, further studies are needed at a genomic, transcriptomic, proteomic, and metabolomic level to explore the mechanisms of Zn-NPs in inducing the general enhancement in physiological functions that, especially under SS, appear a valuable means to face stress. Lastly, more investigations are needed to optimize the rate of Zn-NPs for the wheat crop under variable cropping systems and climate conditions.

## Data availability statement

The original contributions presented in the study are included in the article/supplementary material.

## Author contributions

MUC and IK: conceptualization. TA: data collection. MUC and MUH: writing original draft. IK, MN, MBC, HMA, RYG, NRA, SA and LB review and editing. All authors contributed to the article and approved the submitted version.

## Funding

This research was funded by the Researchers Support Project (RSP-2021/123), King Saud University, Riyadh, Saudi Arabia.

## Conflict of interest

The authors declare that the research was conducted in the absence of any commercial or financial relationships that could be construed as a potential conflict of interest.

## Publisher’s note

All claims expressed in this article are solely those of the authors and do not necessarily represent those of their affiliated organizations, or those of the publisher, the editors and the reviewers. Any product that may be evaluated in this article, or claim that may be made by its manufacturer, is not guaranteed or endorsed by the publisher.
